# Acute peripheral unilateral vestibulopathy of the whole nerve causes increased impairment of spatial orientation and poorer long-term outcome

**DOI:** 10.1007/s00415-025-13160-7

**Published:** 2025-05-26

**Authors:** Christoph Best, Heidrun H. Krämer, Marianne Dieterich

**Affiliations:** 1 Department of Neurology, Philipps-University, Baldingerstrasse, 35043 Marburg, Germany; 2 Department of Neurology, Johannes Gutenberg-University, Mainz, Germany; 3 Department of Neurology, Justus Liebig University, Giessen, Germany; 4 Department of Neurology, LMU University Hospital, Munich, Germany; 5 German Center for Vertigo and Balance Disorders, LMU University Hospital, Munich, Germany

**Keywords:** Acute peripheral unilateral vestibulopathy, UVP, Inferior vestibular nerve, Spatial orientation, Psychological distress, Vestibular neuritis

## Abstract

**Background:**

Acute peripheral unilateral vestibulopathy (UVP) is the third most common cause of peripheral vestibular vertigo. Etiologically, a viral inflammation is assumed. In most cases, an isolated dysfunction of the superior part of the vestibular nerve can be found (superior part UVP = sUVP), but an additional involvement of the inferior part has also been shown (whole nerve UVP = s+iUVP). The aim of the study was (a) to determine the prevalence of an additional inferior part involvement, (b) to quantify the extent of vestibular deficit comparing sUVP vs. s+iUVP and (c) to examine the long-term outcome focusing on psychological distress as well as long-lasting symptoms associated with dizziness.

**Methods:**

96 UVP patients were enrolled. They underwent a neuro-otological examination including measurements of cervical vestibular evoked myogenic potentials (cVEMP), subjective visual vertical (SVV), ocular torsion (OT), caloric testing and the clinical head impulse test (HIT) in the acute phase. The Symptom Checklist-90 R and the Vertigo Symptom Scale were examined at a mean follow-up interval of 4.0 years (± 0.4 years) after disease onset.

**Results:**

Among the 96 patients (47 female; mean age 58 ± 14 years), additional involvement of the inferior nerve part was found in 35 cases (36%). These patients showed a significantly greater tilt of SVV (6.3° ± 4.4° vs. 4.2° ± 3.7°; F = 5.581, p = 0.020) and a more pronounced OT (15.1° ± 8.2° vs. 11.3° ± 7.4°; F = 4.770, p = 0.032) in the acute stage of the disease. The proportion of pathological HIT was significantly higher in the s+iUVP group (82.9% vs. 67.2%; Chi-Square = 20.167, p < 0.001). cVEMPs showed significantly decreased amplitude on the affected side (124.8 µV (± 10.3 µV) vs. 408.4 µV (± 26.6 µV); F = 61.911; p < 0.001). At long-term follow-up, the patients with s+iUVP had significantly increased anxiety scores as compared to patients with isolated sUVP (SCL-90 score for anxiety: 48.4 ± 3.8 vs. 41.6 ± 0.5; F = 4.231, p = 0.026).

**Discussion:**

An additional lesion of the inferior part of the vestibular nerve led to increased vestibular dysfunction in acute UVP and might trigger long-lasting symptom persistence. Identifying these patients early might improve the clinical outcome, lead to a faster improvement and prevent secondary psychosomatic symptoms.

## Introduction

Acute peripheral unilateral vestibulopathy (UVP)—also known as vestibular neuritis—causes a loss or significant reduction of peripheral vestibular function due to an inflammation of the labyrinth or vestibular nerve. Symptoms often appear with an acute or subacute onset of rotary vertigo for days or weeks, nystagmus, impaired balance and a tendency to fall towards the affected ear, nausea and vomiting. The cause is most likely a viral infection (HSV I) [5, 30, 34].

UVP resembles the third most common cause of peripheral vestibular vertigo and the most common cause of an acute vestibular syndrome (AVS) with an annual incidence of 3.5–15.5 per 100,000 and a recurrence rate between 1.9 and 10.7% [1, 22, 26, 35, 37, 38]. Early administration of corticosteroids may improve the recovery [24]. However, a recovery of the peripheral vestibular function appears only in up to 2/3 of the patients [39]. In up to 43% of patients, subjective symptoms still persist after a period of 10 years [33]. It is unclear which factors are causal for this persistence of symptoms associated with subjective vertigo.

The vestibular system has five organs in the labyrinth: the three semicircular canals (anterior, horizontal, posterior) and the two otolith organs (utricle and saccule). The maculae of the utricle and saccule process information on head position in relation to gravity and linear accelerations. The innervation of these five organs is organized via two branches of the vestibular nerve: the superior part innervates the anterior and horizontal canals and the utricle, and a smaller part of the saccular afferents; the inferior part innervates the posterior canal and the majority of the saccular afferents [18, 29, 38, 45].

Clinical routine diagnostics focus on horizontal semicircular canal function using the head impulse test (HIT) and caloric irrigation. It is often unclear which part of the vestibular nerve is involved. So, a diagnostic classification into superior part UVP (sUVP), inferior part UVP (iUVP) or whole nerve UVP (s+iUVP) is not implemented.

Studies of lesion distribution in UVP are few and analysis is limited. Aw and co-workers studied 29 patients with vestibular neuritis [2] and found that 8 patients had deficits in all three semicircular canals, suggesting nerve superior and inferior involvement in 28% of patients. Halmagyi and co-workers reported on two cases [18], while Monstad and co-workers reported on three cases [29], demonstrating typical UVP symptoms but revealing isolated inferior nerve lesions, iUVP, in all cases. The most extensive study, on 43 patients with UVP, found the majority (55.8%) suffered from a combined superior and inferior nerve involvement [40].

In the current study, we addressed two questions: (a) do patients with different vestibular lesion patterns (sUVP vs. s+iUVP) show different clinical presentations especially focusing on spatial orientation and roll plane function and (b) does the long term outcome of these two UVP groups differ, characterizing a possible explanation for subjective symptom persistence even years after the acute stage of the disease?

## Methods

### Study design and participants

In this monocentric prospective study, patients were enrolled after the diagnosis of an UVP was established according to the diagnostic criteria published in the consensus document of the Bárány Society [36]. The following six criteria had to be fulfilled: acute or subacute vertigo, spontaneous vestibular nystagmus, reduced function of the vestibulo-ocular reflex (VOR), no central neurological symptoms, no audiological or otological symptoms, symptoms not better explained by any other disease [36]. Patients were included within 72 h after transfer to the University Hospital of Mainz, Germany, for diagnostic and therapeutic procedures for their underlying vertigo disease. All participants gave their informed written consent. The study was approved by the local ethical committee and performed in accordance with the standards of the Declaration of Helsinki.

### Diagnosis of involvement of the inferior part of the vestibular nerve

In contrast to the clearly defined diagnostic criteria of UVP [36], no such clear criteria exist for differentiation and characterization of the involvement of the superior or inferior part of the vestibular nerve. A few studies defined the additional lesion of the inferior part by pathological cervical vestibular evoked myogenic potentials (cVEMP) [2, 18, 25, 29, 40]. In accordance with these studies, a cVEMP examination was classified as pathological, if the difference in amplitude between both ears exceeded 50%.

### Neurological and neuro-otological diagnostic procedures

Each patient underwent a detailed neurological and neuro-otological examination in the acute phase including positioning maneuvers, gait and stance analysis, clinical head impulse test (HIT) for the horizontal semicircular canals, examination with Frenzel’s glasses and a head shaking test. A funduscopy using a scanning laser ophthalmoscope to determine the torsional eye position, and an examination of a tilt of the subjective visual vertical (SVV) for verticality and spatial orientation were performed to detect an impairment of the graviceptive vestibular function. Electrooculography (EOG) including caloric stimulation was used to test the function of the horizontal semicircular canals. The following parameters were selected: unilateral hyporesponsiveness in bithermal caloric test, tilting of the SVV, torsion of the eyes (OT), pathological HIT and cVEMPs.

#### Caloric vestibular testing

Unilateral hyporesponsiveness was calculated according to Jongkees’ formula using the velocity of the slow nystagmus phase: {[(R30° + R44°) − (L30° + L44°)]/[R30° + R44° + L30° + L44°]} × 100 in percent. Thus, vestibular hyporesponsiveness as detected by caloric irrigation was defined as a side asymmetry of at least 25% according to Honrubia’s recommendation [21].

#### Ocular torsion

To determine the extent of ocular torsion and thus the roll plane function as well as the otolith function, the angle between a straight line through the optic papilla and macula relative to the horizontal was calculated as the mean value of six examinations. The images of the papilla-macula line position were taken with a scanning laser ophthalmoscope. Normal eye torsion and position is an excyclotropy of approximately 5° (for details see [11, 14]). Deviations of more than 8° between the torsion/position of both eyes were defined as pathologic.

#### Static subjective visual vertical

Tilts of SVV were examined for graviceptive (utricular) dysfunction. Patients were seated in an upright position and looked into the static center of a hemispherical dome that could be rotated around the patient’s visual axis. The surface of the dome was covered with a randomized dot pattern giving no visual indication of gravitational orientation. The patients had to align a target line from a random offset position in the center of the dome vertically. After each measurement the dome was rotated to avoid visual help. The result was calculated as the mean value from seven individual measurements. The physiological range of SVV under static visual conditions is ± 2.5° [13–15, 19].

#### Cervical vestibular evoked potentials

cVEMPs were recorded from the pre-innervated sternocleidomastoid muscle using surface electrodes (AG/AGCl, Hoerniß and Zeisberg). Auditory stimulation was monaural and ipsilateral to the pre-innervated muscle. Square wave click sounds (0.1 ms duration, 95 dB above hearing threshold) were applied as an acoustic stimulus with at least 128 repetitions.

P13/N23 amplitudes of less than 100 μV or absent response amplitudes were defined as pathological, and an amplitude difference that exceeded 50% of the absolute amplitude of the smaller P13/N23 response was considered to be pathological. The recording was performed on a Neuroscreen workstation (Jaeger/Toennies). The examination of cVEMPs revealed significantly reduced P13/N23 amplitudes in s+iUVP patients on the involved side (sUVP vs. s+iUVP: 408.4 µV (± 26.6 µV) vs. 124.8 µV (± 10.3 µV); F = 61.911, p < 0.001). No difference of cVEMP amplitudes was detected for the unaffected side. The relative side difference in P13/N23 amplitudes was significantly increased in s+iUVP patients (sUVP vs. s+iUVP: 22.9% (± 1.8%) vs. 63.2% (± 2.2%); F = 193.244, p < 0.001) (Table 1).

**Table 1:** Demographic, clinical, neuro-otological and long-term data

	sUVP (n = 61)	s+iUVP (n = 35)	F- and p-value
Age, yrs, mean (SD)	55.1 (± 15.4)	59.8 (± 10.9)	n.s
Female patients (%)	25 (41)	22 (63)	F = 4.363; p = 0.039
Neurological results			
Caloric hyporesponsiveness (%)	45.8 (± 32.4)	45.5 (± 33.6)	n.s
Ocular torsion in degrees (SD)	11.3 (± 7.4)	**15.1 (**± **8.2)**	F = 4.770; p = 0.032
Subjective visual vertical in degrees (SD)	4.2 (± 3.7)	**6.3 (**± **4.4)**	F = 5.581; p = 0.020
Pathological HIT (%)	67.2%	**82.9%**	Chi = 20.167, p < 0.001
cVEMP			
P13/N23 amplitude affected side [µV]	408.4 (± 26.6)	**124.8 (± 10.3)**	F = 61.9; p < 0.001
P13/N23 amplitude healthy side [µV]	404.1 (± 24.1)	372.5 (± 10.3)	n.s.
Side difference in [%]	22.9 (± 1.8)	**63.2 (± 2.2)**	F = 193.2, p < 0.001
Psychological long term results			
Vertigo-associated anxiety (SCL 90) (SD)	41.6 (± 1.5)	**48.4 (**± **3.8)**	F = 4.231; p = 0.026
Severity of symptoms (SCL 90) (SD)	40.8 (± 1.3)	43.6 (± 2.1)	n.s.
Vertigo symptom scale anxiety (VSS-A) (SD)	11.6 (± 1.6)	15.2 (± 1.9)	n.s.
Vertigo symptom scale severity (VSS-S) (SD)	6.0 (± 1.9)	8.6 (± 1.2)	n.s.

Distribution of age did not differ between groups, the proportion of female patients, however, was larger in s+iUVP. No significant differences were found for caloric hyporesponsiveness. Ocular torsion and subjective visual vertical showed significantly larger displacements in s+iUVP. HIT was pathological in a significantly greater proportion in s+iUVP. In s+iUVP, cVEMPs on the lesioned side were significantly reduced in P13/N23 amplitude. The side difference of cVEMPs was significantly increased in s+iUVP. On long-term follow-up, the psychometric evaluation by various parameters revealed elevated psychological distress for patients with s+iUVP

*cVEMP* cervical vestibular-evoked myogenic potential, *HIT* head impulse test, SCL 90 Symptom Checklist 90, *SD* standard deviation, *sUVP* unilateral vestibulopathy of the superior nerve part, *s+iUVP* whole nerve unilateral vestibulopathy, superior and inferior nerve parts, *VSS-A* Vertigo Symptom Scale, vertigo associated anxiety, *VSS-S* Vertigo Symptom Scale, severity of symptoms

### Psychometric evaluation during follow-up

All patients were contacted and invited to participate in a long-term follow-up by psychometric evaluation focusing on their psychological wellbeing and symptoms associated with subjective vertigo. The Vertigo-Symptom-Scale (VSS) and the Symptom Checklist 90 R (SCL-90 R) were used as psychometric instruments to reveal psychosocial distress in relation to their past vertigo disorder.

#### VSS

The VSS assesses symptoms of dysfunction of the posture and balance system such as unsteadiness, dizziness, vertigo and postural instability. Symptoms like somatic anxiety (VSS-A) and vertigo associated symptom severity (VSS-S) were examined. Thus, the severity of the different aspects of dizziness-associated symptoms was classified [42, 44].

#### SCL-90 R

The Symptom Checklist 90 Revised (SCL-90 R) assesses psychological symptoms and distress using a 90-item self-rated questionnaire. It aims to classify psychological distress into distinct symptom categories, such as somatization, obsessive–compulsive, interpersonal sensitivity (e.g., [41]).

By application of these two psychometric tools, a broad range of psychological symptoms and distress can be covered.

### Statistical analyses

All statistical analyses were performed using SPSS 29 (IBM, CA, USA). A univariate ANOVA was applied to analyze the difference between the two patient groups, sUVP vs. s+iUVP, after a normal distribution of data was confirmed. Chi-square analysis was used for dichotomous variables. Data are presented as mean ± standard error for parametric data, if not quoted differently. A p-value < 0.05 was considered significant.

## Results

### Demographic data and clinical findings in the acute phase:

**Fig. 1:**
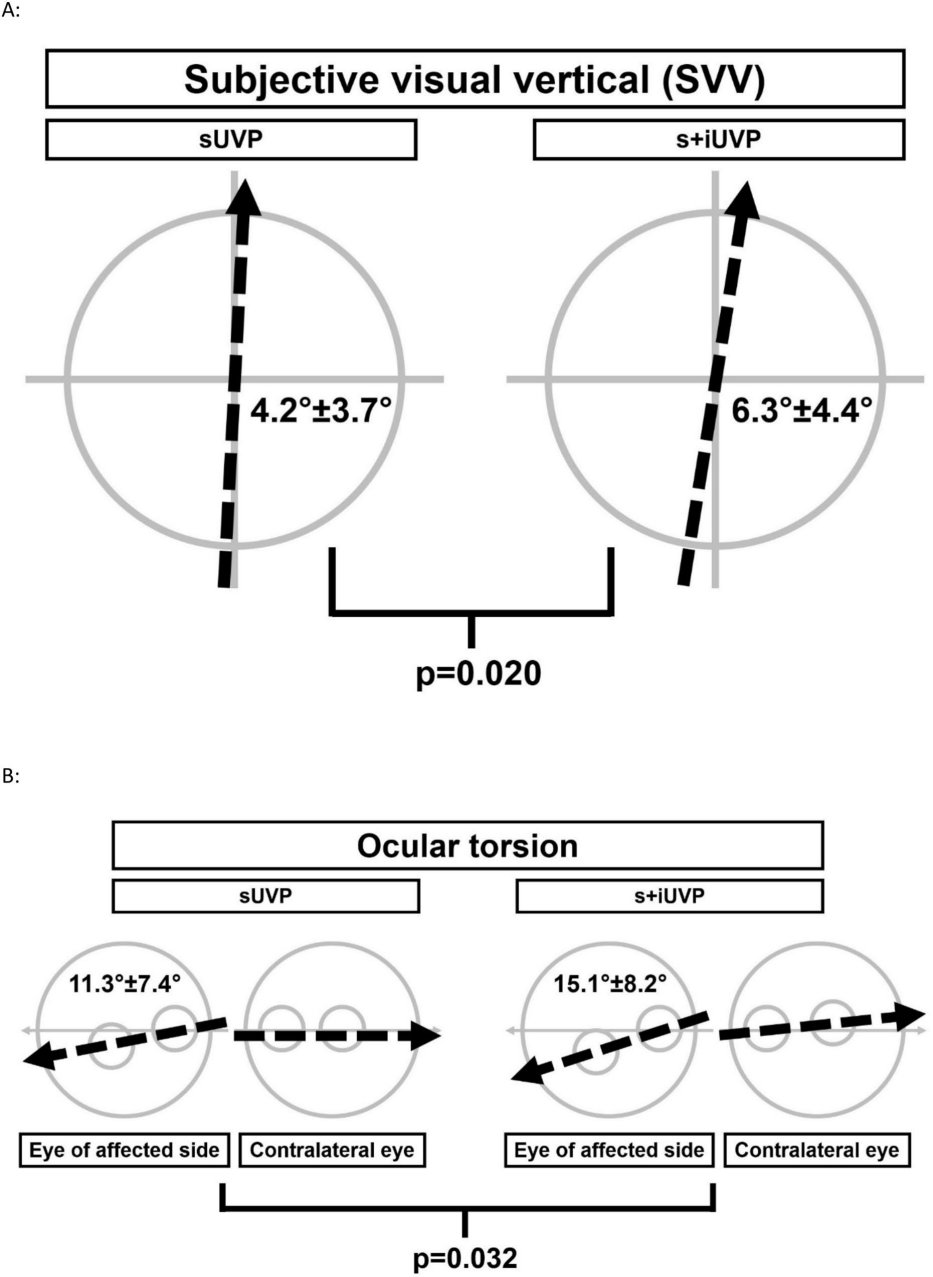
**A** Depicts a schematic illustration of the significantly greater tilt of the SVV (6.3 ± 4.4 vs. 4.2 ± 3.7; F = 5.581, p = 0.020). The intersected arrows represent the mean value of the group results, comparing patients with an isolated sUVP on the left side with s+iUVP patients on the right side, revealing a significantly greater SVV displacement. **B** Presents the significantly greater OT of the patient group with s+iUVP (15.1 ± 8.2 vs. 11.3 ± 7.4; F = 4.770, p = 0.032) on the right side, patients with an isolated sUVP are illustrated on the left side. The intersected arrows present the mean value of the group results. The large grey circle shows the ocular fundus, the two small grey circles represent macula and optic papilla, while the small circle in the peripheral field of the fundus shows the optic papilla and the small circle located at the horizontal midline shows the macula. *sUVP* nilateral vestibulopathy of the superior nerve part, *s+iUVP* whole nerve unilateral vestibulopathy, superior and inferior nerve parts

96 patients with acute UVP were prospectively enrolled in the study (47 female, 49 male; mean age 58 ± 14 years). Distribution of age did not differ between the patient groups (sUVP vs. s+iUVP), the proportion of female patients, however, was larger in the s+iUVP group (F = 4.363, p = 0.039). No significant differences were found concerning the unilateral caloric hyporesponsiveness in the context of the underlying UVP. Of those 96 patients, a total of n = 35 (36%) presented with a combined involvement of the superior and the inferior part of the vestibular nerve resulting in s+iUVP. In 61 patients (64%), an isolated lesion of the superior part was detected. We did not identify patients with an isolated lesion of the inferior part of the vestibular nerve. In the acute phase of UVP, patients with s+iUVP showed a significantly greater tilt of the SVV and a significantly increased OT (for details, see Fig. 1). Overall, measurements of spatial orientation and vestibular roll plane function (SVV tilt and OT) were significantly more impaired in the s+iUVP patients.

Performing a clinical HIT, 41 out of 61 sUVP patients (67.2%) had a pathological HIT, while 29 out of 35 s+iUVP patients (82.9%) presented with a pathological HIT. The difference between the groups was significant (Table 1).

### Psychometric results at long-term follow-up

Of the 96 patients initially included, a total of 49 agreed to participate in the follow-up examination using psychometric interviews. The mean follow-up interval was 4.0 years (± 0.4 years).

#### SCL-90 R

**Fig. 2:**
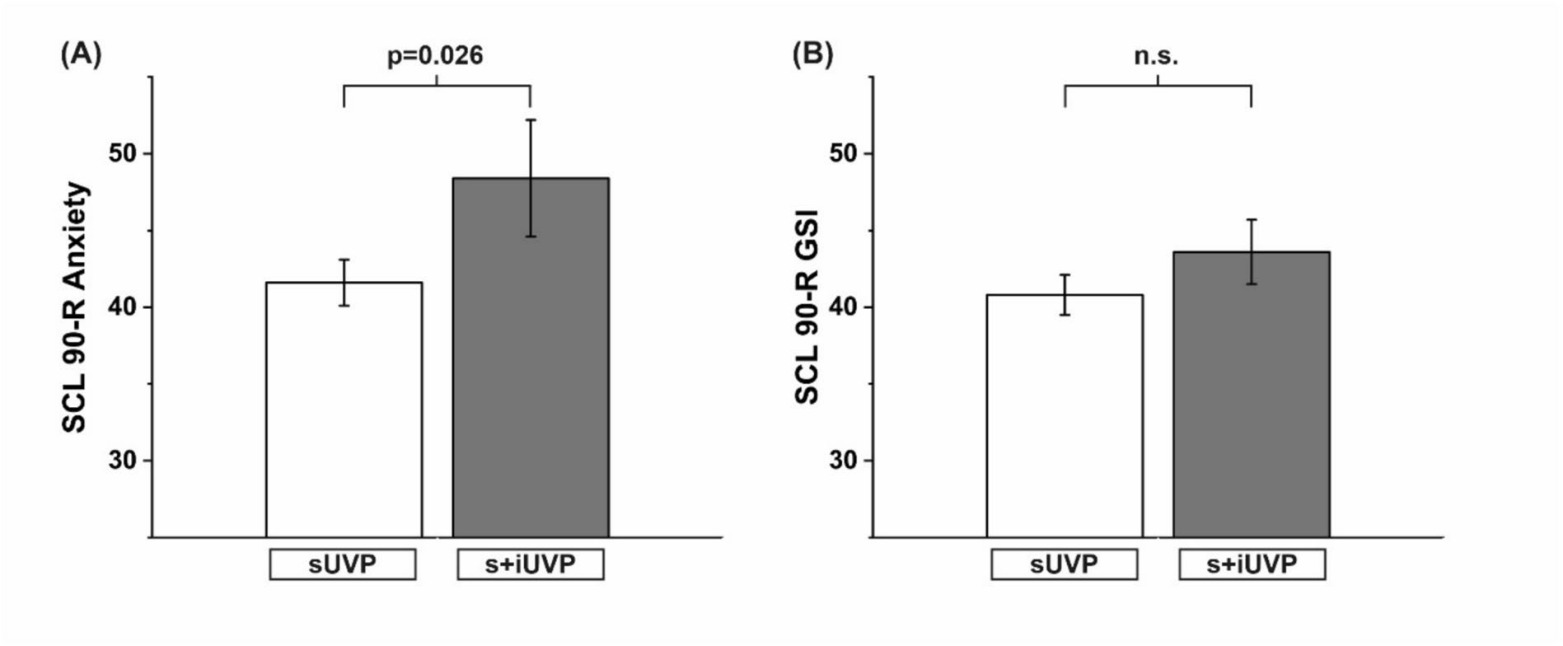
**A** Greater psychological distress by revealing significantly elevated values for anxiety in patients with s+iUVP as compared to patients with an isolated sUVP (anxiety values: 48.4 ± 4.8 vs. 41.6 ± 1.5; F = 4.231, p = 0.026). **B** Elevated values for the severity of s+iUVP patients are displayed; these failed to become significant (SCL-90 R; GSI: 43.6 ± 2.1 vs. 40.8 ± 1.3). *GSI* Global severity index, as subscale of SCL 90, *SCL 90* Symptom Checklist 90, *sUVP* unilateral vestibulopathy of the superior nerve part, *s+iUVP* whole nerve unilateral vestibulopathy, superior and inferior nerve parts

Applying the SCL-90 R as a psychometric self-reporting instrument measuring psychic strain, patients with initial s+iUVP had significantly higher values of disease-associated anxiety. Also the scale for disease-associated severity of experienced symptoms revealed elevated values in the group of patients with s+iUVP (For details, see Fig. 2).

#### VSS

VSS-A: Applying the vertigo symptom scale to evaluate the subjective ratings of anxiety caused by vertigo and dizziness showed distinctly elevated results in the group of s+iUVP patients but the level of significance was not met.

**Fig. 3:**
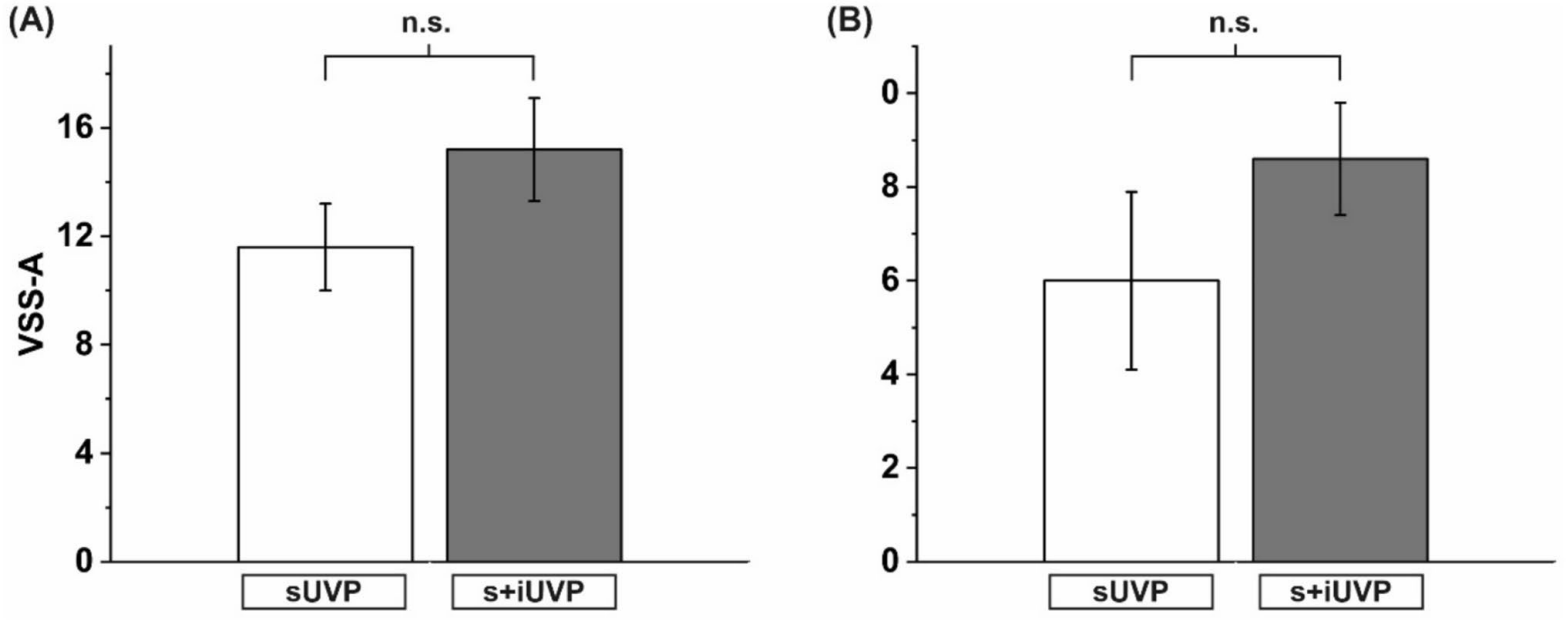
**A** Elevated psychological distress in patients with s+iUVP is demonstrated by elevated values of anxiety applying the VSS in its subscale “anxiety” as compared to patients with isolated sUVP. **B** Depicts elevated psychological distress in s+iUVP patients applying the VSS in its subscale symptom severity as compared to patients with isolated sUVP. *sUVP* unilateral vestibulopathy of the superior nerve part, *s+iUVP* whole nerve unilateral vestibulopathy, superior and inferior nerve parts, *VSS-A* Vertigo Symptom Scale, vertigo associated anxiety, *VSS-S* Vertigo Symptom Scale, severity of symptoms

VSS-S: The subjective perceived severity of vertigo and dizziness-associated symptoms showed higher values in the patients with s+iUVP compared to the patients with sUVP but the level of significance was also not met. Again, results demonstrating an elevated severity of symptoms as already gained by the SCL-90 R could be reproduced by a second psychometric tool (Fig. 3). To summarize, patients who had suffered from s+iUVP presented 4 years later with increased vertigo-associated anxiety and severity of experienced symptoms as compared to patients with an isolated sUVP.

## Discussion

In this large cohort of 96 patients with acute UVP, systematically analyzed with respect to the individual involvement of the superior part and the inferior part of the vestibular nerve, an isolated lesion of the superior part was found in 64%. In 36% of the patients, evidence for a combined involvement, a lesion of the whole nerve (s+iUVP), was disclosed. Patients with an isolated lesion of the inferior part of the vestibular nerve were not identified. The patients with s+iUVP showed significantly increased tilts of SVV and significantly increased OT, representing a remarkably stronger disturbance of spatial orientation and roll plane function. This was due to the simultaneous damage of posterior semicircular canal and otolith (mainly utricular) function. They also revealed a significantly higher proportion of pathological HIT and reduced cVEMP amplitudes. The latter was due to the lesion of saccular function. Applying psychometric evaluation during long-term follow-up after 4 years, patients with s+iUVP presented with higher levels of symptom-related distress and anxiety.

### Distribution of involvement in sUVP, iUVP or s+iUVP

Regarding the rate of involvement of both parts of the vestibular nerve or the superior nerve part only, our data fit well with the few previous findings. In 29 patients with UVP, an isolated lesion of the superior part was found in 72% (21 out of 29 patients) and a combined involvement in 28% (8 out of 29 patients) [2]. In two case reports with additional hearing loss, an isolated lesion of the inferior part of vestibular nerve was discussed [2]. In the larger group of 43 UVP patients, evidence for a combined involvement of the superior and inferior parts was found in 55.8% (24 out of 43), an isolated lesion of the superior part in 41.9% (18 out of 43) and an isolated lesion of the inferior part in one case only [40]. Out of 703 patients with the diagnosis of UVP, Kim and Kim detected a total of 9 patients with the diagnosis of isolated iUVP, corresponding to 1.3% of the patients [25]. Thus, an isolated lesion of the inferior part of the vestibular nerve is rare.

The prevalence for an isolated involvement of the superior nerve part varied between 41.9% and 72% of UVP patients. A combined whole nerve lesion showed a prevalence between 28% and 55.8%. This broad scattering of prevalence is possibly due to the limited number of patients in a small number of studies. Therefore, our findings of an isolated lesion of the superior nerve part in 64% and a whole nerve involvement in 36% of UVP patients were in agreement. We did not detect any isolated lesion of the inferior nerve part, which was also in accordance with previous studies in which this was found in a rare 1.3% [25].

There are three possible explanations for such a distribution of lesion pattern and as to why the inferior nerve part is often spared in UVP: (a) the bony canal for the passage of the superior part of the vestibular nerve is smaller [17] and therefore responsible for a stronger damage by swelling of the nerve in the acute phase of inflammation, (b) the posterior semicircular canal has a double and redundant innervation [10], and (c) anastomoses between the facial nerve and the superior nerve part of the vestibular nerve may cause a spreading of HSV I virus beginning with the superior branch [10].

### Spatial orientation and roll plane dysfunction

Detailed testing of all five vestibular endorgans analyzing the clinical consequences of involvement of the different nerve parts in UVP is, as mentioned above, rare. Most studies focused on specific aspects. High rates of disturbances in spatial orientation with 90% pathological results for subjective visual horizontal were detected in 36 patients out of a cohort of 40 UVP patients [40]. However, in the latter study a differentiated analysis comparing superior nerve, inferior nerve, and whole nerve involvement focusing on verticality perception was not performed. Furthermore, measurements of ocular torsion—indicating a dysfunction of graviceptive vestibular pathways in the roll plane of the VOR achieved mainly by utricular otolith and vertical posterior semicircular canal input—were not carried out. Other studies reported on inferior nerve part involvement but did not perform measurements of verticality or ocular torsion [2, 18, 29]. Thus, we present a large cohort addressing the questions of a disturbance of the superior and/or inferior nerve part UVP by performing a differentiated analysis of the function of the horizontal canals (calorics, HIT) and the utricle (SVV, OT) for the superior branch and additionally of the posterior canals (SVV, OT) and the saccule (cVEMPs) for the inferior branch.

### Course of recovery and compensation

In a prospective study on rehabilitation in patients with a unilateral vestibular hypofunction, a subgroup of about 20% did not benefit from the vestibular rehabilitation [20]. The reason for such a subjective symptom persistence [33] in about 20–40% of the patients with chronic UVP has not been fully understood. Recovery after an UVP is based on three main mechanisms: (a) recovery of peripheral vestibular function, (b) somatosensory and visual substitution, and (c) central compensation within the bilateral multisensory networks. Sensory substitution and central compensation are achieved by vestibular training and rehabilitation [37]. Van Laer and colleagues identified that poor static balance performance was a risk factor for chronic dizziness even in a cohort receiving vestibular rehabilitation [43]. Lacour and colleagues found that static and dynamic vestibular deficits have different courses of recovery. [27]. The static vestibular deficits are due to lesions of the horizontal and vertical canal and utricular afferents in the low-frequency range which are compensated after a longer time constant of several months and up to a year. However, the dynamic vestibular deficits of the higher frequency ranges are not well compensated due to the permanent decrease in gain and phase shift of the VOR [27]. Hwang and coworkers performed a follow-up on 46 patients with UVP and divided them into sUVP and s+iUVP. At a 6–12 month follow-up, 65% of sUVP patients presented with good recovery while only 20% of the patients with s+iUVP showed such a beneficial course [23]. The authors concluded that a larger extent of nerve involvement (s+iUVP) correlated with a worsening of outcome. These data, however, were limited to analyses of semicircular canal paresis and neglected the otoliths. In a retrospective chart analysis of 59 acute UVP patients, Navari and colleagues suggested that s+iUVP might cause an unfavorable outcome as compared to sUVP [31]. In s+iUVP, 11 out of 19 patients (58%) did not show spontaneous clinical recovery and had to undergo vestibular rehabilitation, whereas in sUVP it was 8 out of 27 patients (30%) [31]. Perception of verticality and graviceptive roll plane dysfunction had not been not evaluated.

These assumptions of an unfavorable outcome in whole nerve lesions are in agreement with the data of the current study in chronic UVP: 20–40% of unexplained persistence of subjective symptoms in combination with an unfavorable course of our long-term data showing increased psychological distress and anxiety in s+iUVP, compared to isolated sUVP, further pinpoints the reason for subjective symptom persistence towards the additional involvement of the inferior vestibular nerve afferents.

### Psychological distress at follow-up

Psychological distress and psychiatric comorbidity have been reported with vestibular disorders [7, 16, 28]. The course of the disease, whether unpredictable or controllable, affects the development of these phenomena [6, 16]. The extent of vestibular function also plays a crucial role in chronic vestibular disorders. Decker and co-workers analyzed vertigo-related anxiety and psychiatric comorbidity in 7083 chronic dizzy patients [12]. They concluded that a functioning vestibular system is necessary for developing vertigo-related anxiety and subsequent psychiatric impairment. Bilateral vestibulopathy did not result in elevated rates of secondary impairments, despite falls being more frequent [12]. Higher anxiety levels were associated with excited vestibular system activity (vestibular migraine, functional dizziness), while reduced anxiety was associated with diminished vestibular function [9, 12]. A recent fMRI meta-analysis comparing brain activations during vestibular stimulation and fear conditioning confirmed the close interaction between the vestibular system and the anxiety/emotional systems [32]. The authors proposed a concept of unfamiliar vestibular stimulations or acute vestibular disorders making unpleasant perceived body accelerations and nausea less distressing by down-regulating the fear network [32]. This hypothesis was based on the differential effects of galvanic stimulation (activation) and fear conditioning (deactivation) to the activity of the posterior insula in fMRI and corresponds to clinical observations in patients with bilateral vestibulopathy. A possible explanation therefore is given by Balaban and colleagues [4]. They propose that balance and vestibular disorders and psychiatric comorbidities (e.g. psychological distress) are linked. They suggest six neuronal circuits/networks that process vestibular, autonomic, emotional and pain information, e.g. the afferent interoceptive information processing complex and the raphe–locus coeruleus loop [3].

In the current study, we compared sUVP and s+iUVP patients using psychometric evaluation after four years. We found elevated anxiety and symptoms associated with vertigo in s+iUVP. Initially, this may be in contrast to findings that poorer bilateral (and probably also unilateral) vestibular function correlates with less anxiety. In a one-year follow-up, we showed that patients with UVP had better psychological recovery over one year than those with vestibular migraine, Menière's disease or benign paroxysmal positional vertigo [7, 8]. Nonetheless, these secondary psychological findings were elevated in UVP patients in the acute stage and most had a lesion of the superior part of the nerve. Now, we analyzed UVP patients at an even later follow-up and 27% had a deficit of the whole nerve. In trying to understand why 20–40% of UVP patients still have chronic symptoms despite a prognostically positive disease course, the additional involvement of the inferior part of the vestibular nerve may be part of the answer.

### Limitations

Our study had some limitations. One is the fact that patients at follow-up only received psychometric interviews and that a second neurological or neuro-otological examination including the vestibular tests was not performed. Especially focusing on the inferior vestibular nerve lesion as a potential risk factor for an unfavorable outcome, such a design for a prospective study including a detailed neuro-otological examination is recommended. Furthermore, diagnosis of unilateral vestibular hyporesponsiveness was made on the basis of a caloric irrigation, therefore examining the vestibular function of the horizontal canals in the low-frequency spectrum. A video head impulse test (vHIT) was not performed, only a clinical HIT for the horizontal semicircular canal, analyzing the vestibular function in the high-frequency range with clinical data but without quantitative results. The absence of a vHIT, and consequently the absence of analysis of posterior canal function, may have resulted in an underestimation of the overall proportion of patients with s+iUVP. Taylor and colleagues demonstrated that, in certain instances, the presence of an additional inferior vestibular nerve involvement during UVP could result in a discordant pattern of function tests for posterior semicircular canal and the otolith function. Consequently, in 15 out of 41 patients, contradictory results were obtained from the vHIT (PC) and cVEMP analyses. In eight of the 41 patients, pathological cVEMPs were observed in conjunction with normal PC vHIT. In 7 out of 41 patients (17%), the cVEMPs yielded normal results, whereas the PC vHIT analysis for the posterior semicircular canal revealed a pathological finding [40]. In view of the foregoing, a discussion is required regarding the proportion of patients with additional involvement of the inferior vestibular nerve, and whether this proportion exceeds the currently calculated 36%.

Moreover, patients received testing of hearing threshold before conducting cVEMPs, but an audiogram was not documented. In addition, a possible bias might be the reduced number of 49 patients who agreed to participate in the follow up interviews. These might represent patients with persisting vestibular deficits and symptoms.

## Conclusion

UVP can involve the whole vestibular nerve as well as the superior or inferior nerve parts in isolation. Isolated superior nerve part involvement was most common (up 72% of patients), the involvement of the whole nerve was still reported often (up to 55.8%), and the separate lesion of the inferior part appeared rarely. Patients with whole nerve involvement showed greater impairment of spatial orientation (verticality perception) and of roll plane function in the acute stage, while they revealed higher levels of disease-related anxiety and psychological distress in the chronic phase compared to patients with a lesion of the superior part only.

## Abbreviations

ANOVA: Analyses of variance
AVS: Acute vestibular syndrome
cVEMP: Cervical vestibular evoked myogenic potential
EOG: Electrooculography
HIT: Head impulse test
HSV I: Herpes simplex virus type I
iUVP: Inferior nerve part unilateral vestibulopathy
OT: Ocular torsion
SCL-90 R: Symptom Checklist 90 R
SD: Standard deviation
SVV: Subjective visual vertical
sUVP: Unilateral vestibulopathy of the superior nerve part
s+iUVP: Whole nerve unilateral vestibulopathy, superior and inferior nerve parts
UVP: Unilateral vestibulopathy
VOR: Vestibulo-ocular reflex
VSS: Vertigo Symptom Scale
